# Identification of AflR Binding Sites in the Genome of *Aspergillus flavus* by ChIP-Seq

**DOI:** 10.3390/jof6020052

**Published:** 2020-04-21

**Authors:** Qing Kong, Perng-Kuang Chang, Chunjuan Li, Zhaorong Hu, Mei Zheng, Quanxi Sun, Shihua Shan

**Affiliations:** 1 School of Food Science and Engineering, Ocean University of China, Qingdao 266003, China; kongqing@ouc.edu.cn; 2 Southern Regional Research Center, Agricultural Research Service, US Department of Agriculture, New Orleans, LA 70124, USA; perngkuang.chang@usda.gov; 3 Shandong Peanut Research Institute, Qingdao 266110, China; peanutlab@163.com (C.L.); squanxi@163.com (Q.S.); 4 State Key Laboratory for Agrobiotechnology and Key Laboratory of Crop Heterosis and Utilization (MOE), Department of Plant Genetics and Breeding, China Agricultural University, Beijing 100193, China; zrhu@cau.edu.cn (Z.H.); zhengmei415@126.com (M.Z.)

**Keywords:** AflR, *Aspergillus flavus*, ChIP-seq, binding motif, gene regulation

## Abstract

We report here the AflR binding motif of *Aspergillus flavus* for the first time with the aid of ChIP-seq analysis. Of the 540 peak sequences associated with AflR binding events, 66.8% were located within 2 kb upstream (promoter region) of translational start sites. The identified 18-bp binding motif was a perfect palindromic sequence, 5′-CSSGGGWTCGAWCCCSSG’3′ with S representing G or C and W representing A or T. On closer examination, we hypothesized that the 18-bp motif sequence identified contained two identical parts (here called motif A and motif B). Motif A was in positions 8–18 on the upper strand, while motif B was in positions 11-1 on the bottom strand. The inferred length and sequence of the putative motif identified in *A. flavus* were similar to previous findings in *A. parasiticus* and *A. nidulans*. Gene ontology analysis indicated that AflR bound to other genes outside the aflatoxin biosynthetic gene cluster.

## 1. Introduction

The biosynthesis of the potent carcinogen, aflatoxin (AF), has been studied extensively and the biosynthetic gene cluster has been identified [1,2]. Molecular analysis has indicated that the transcription factor AflR/Afl-2, which contains a GAL4-type binuclear zinc finger cluster domain, CX2CX6CX6CX2CX6CX2, plays a key role in AF biosynthesis [3,4]. To better understand the function of AflR, it is very important to study the binding motif and specificity of AflR. Fernandes et al. have demonstrated that *Aspergillus nidulans* AflR binds to the palindromic sequence 5′-TCG(N5)CGA-3′ in the promoter of the *stcU* gene involved in sterigmatocystin biosynthesis [5], and Ehrlich et al. have reported the AflR binding site (5′-TCGSWNNSCGR-3′) in promoter regions of several AF biosynthetic genes of *A. parasiticus* [6]. However, the AflR binding motif in *A. flavus* has not been reported yet. The *A. flavus* AflR is around 99% and 33% identical to *A. parasiticus* AflR and *A. nidulans* AflR, respectively [7]. Although AflR is conserved in closely related aspergilli, it is likely that there will be some degeneracy in binding specificity of respective AflRs. Furthermore, the known AflR binding motifs were identified by the aid of Electrophoretic Mobility Shift Assay (EMSA) in vitro [5,6], while EMSA doesn’t fully reflect the actual situation in vivo.

The genome sequencing of *A. flavus* has been completed [8,9]. The genome size and predicted number of genes of *A. flavus* are 36.8 Mb and 12,197, respectively. The number of AflR binding sites in the *A. flavus* genome would be expected to be about 2211 by chance, based on the length (11 bp) of the AflR binding motif sequences of *A. parasiticus* and *A. nidulans*. Price et al. first reported that AflR regulates the expression of three genes (*nadA*, *hlyC*, and *niiA*) outside of the AF biosynthetic cluster under conditions conducive to AF production in *A. parasiticus* [10]. Their data show that AflR may have a broad function and regulates other genes in addition to genes in the AF gene cluster. The cDNA microarray which Price et al. used represents about 40% of the *A. flavus* transcriptome. With the advent of the genomics era, it may be fruitful to examine the *A. flavus* genome for additional genes to which AflR can bind.

Chromatin immunoprecipitation followed by sequencing (ChIP-seq), which combines chromatin immunoprecipitation (ChIP) and DNA sequencing, is an effective method to study nucleosomes positioning, protein-DNA binding events, or histone modifications on a genome-wide scale [11]. With the decreasing cost of sequencing, ChIP-seq has become an indispensable tool for studying transcription factor binding sites and epigenetic mechanisms [12]. In this research, we report the AflR binding motif of *A. flavus* by the aid of ChIP-seq, and this is the first ChIP-seq report of AflR in *A. flavus*.

## 2. Materials and Methods 

### 2.1. Expression of A. flavus AflR (AfAflR) in Escherichia coli and Production of the Antibody of AfAflR

Expression and purification of AflR (AAM03003.1, NCBI) were carried out according to Fernandes et al. with slight modification [5]. BL21 (DE3) *E. coli* cells were transformed with plasmid pET32a(+) containing *A. flavus aflR*, and a His-tag was used for protein purification [13]. Cells were grown at 37 °C in Luria-Bertani (LB) medium containing 25 μg/mL chloramphenicol and 20 μg/mL kanamycin to an OD_600_ of 0.6. After induction with 1 mM IPTG for 3 h, uninduced and induced cells were harvested and resuspended in 100 mM Tris (pH 7.5). Then, cells were lysed and cell debris was removed by centrifugation. Cell lysates were examined for AfAflR expression by sodium dodecyl sulfate polyacrylamide gel electrophoresis (SDS-PAGE) followed by Coomassie blue staining. AfAflR was purified by metal-chelate affinity chromatography using the Ni-NTA resin from GE Healthcare (Chicago, IL, USA) according to the manufacturer’s instructions. Briefly, a buffer containing 250 mM imidazole was used to elute the samples. 

The multiclonal antibody of AfAflR was produced in rabbits by Abcam (Shanghai, China). Western blot hybridization was used to check the specificity of the antibody [14]. SDS-PAGE and a nitrocellulose filter membrane (Millipore, Merck, Darmstadt, Germany) were used for separating and transferring protein samples. Hybridization was performed by first adding the AfAflR antibody for 2 h followed by adding the goat anti-rabbit antibody (Abmart, Shanghai, China) to the membrane and incubating for 45 min. Signals were detected using the electrochemiluminescence (ECL) detection system (Amersham, Buckinghamshire, England) and photographed by iBright Imaging System (Thermo Fisher Scientific, Waltham, MA, USA).

### 2.2. Chromatin Immunoprecipitation (ChIP)

For ChIP experiments, *A. flavus* NRRL3357 was grown in 200 mL (1 × 10^6^ spores/mL) of potato dextrose broth (PDB) in 500 mL shaking flasks at 28 °C for 24 h [15]. Three replicate cultures were prepared. The cultures were centrifuged and transferred to a cross-linking solution for ChIP experiments. The cross-linking, DNA sonication, and chromatin immunoprecipitation were performed according to the protocols of Chung et al. [16]. Briefly, the chromatin was extracted and sonicated (Branson sonifier, Danbury, CT, USA) at half-maximal power over ten 10-sec pulses with chilling on ice for 2 min after each pulse. An aliquot of the chromatin solution (1/10 of the total volume) was used as input DNA to determine the DNA fragment sizes. The average sizes of the resultant DNA fragments were ~0.2–1.5 kb. The remaining chromatin solution was divided into two parts: one was incubated with the addition of 10 µl of the antibodies (anti-AfAflR), and the other was incubated without antibodies (mock). Immunoprecipitated DNA was used for sequencing. Millipore Chromatin Immunoprecipitation Assay Kit (17-295, EMD Millipore Corporation, Temecula, CA, USA) was used in ChIP experiments.

### 2.3. ChIP Sequencing and Peak Finding

The creation of ChIP-seq libraries, ChIP-sequencing, and peak finding were fulfilled by Berry Genomics (Beijing, China). Briefly, ChIP-sequencing was accomplished on the Illumina HiSeq 2500 with the ChIP-seq libraries [17]. Reads were trimmed and cleaned of Illumina adaptors using Trimmomatic and aligned to the *A. flavus* NRRL3357 genome using bowtie2-2.1.0 [18]. The genome and annotations of *A. flavus* NRRL3357 were downloaded from NCBI (The National Center for Biotechnology Information). Reads that aligned concordantly were used for peak calling. The resulting bam files were used as an input for peak calling by Model-based Analysis for ChIP Sequencing (MACS2) version 2.0.10.20131216 [19]. Peak calling was done with the ChIP-seq samples and input control samples using a False Discovery Rate (FDR) cutoff of 0.05. The topGO R package was used for functional enrichment and gene ontology (GO) analysis as described previously [20,21]. The *p*-value cutoff was set at 0.05 for GO analyses. Results reported herein were the combined reads from the three replicate samples. 

The ChIP-sequencing results were submitted to NCBI’s GEO database and assigned the accession no. GSE149696.

### 2.4. Binding Motif Analysis

The online motif finding program Multiple EM for Motif Elicitation (MEME, Version 5.1.1, available at: http://meme-suite.org/index.html) was used to predict AfAflR-binding motifs within ChIP-seq peaks [22]. The sequences of the 200 bp centered on each of the peaks were uploaded into MEME (Table S1). 

## 3. Results and Discussion

**Figure 1:**
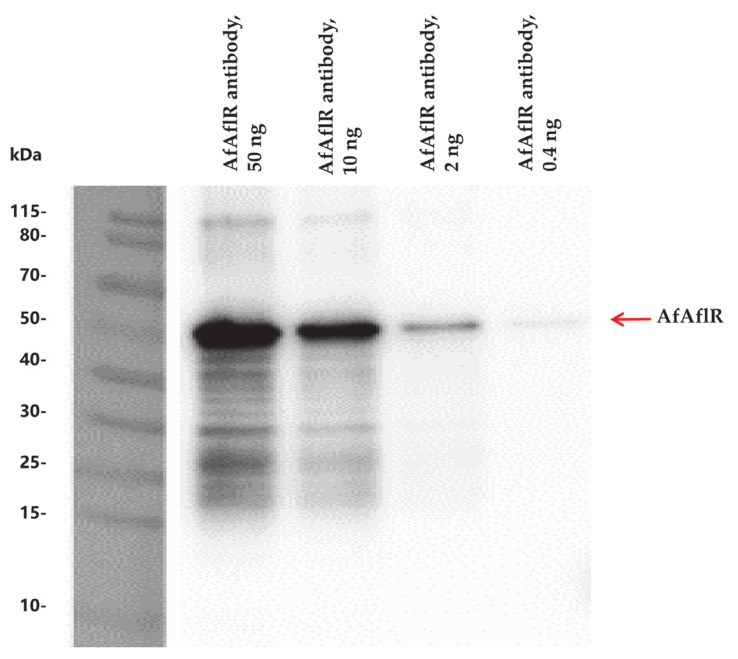
Western blot analysis demonstrating the specificity of the AfAflR antibody.

The molecular weight of recombinant AfAflR was around 50 kDa, and Western blot analysis showed that the multiclonal AfAflR antibody (0.4–50 ng) specifically bound to AfAflR (Figure 1).

**Figure 2:**
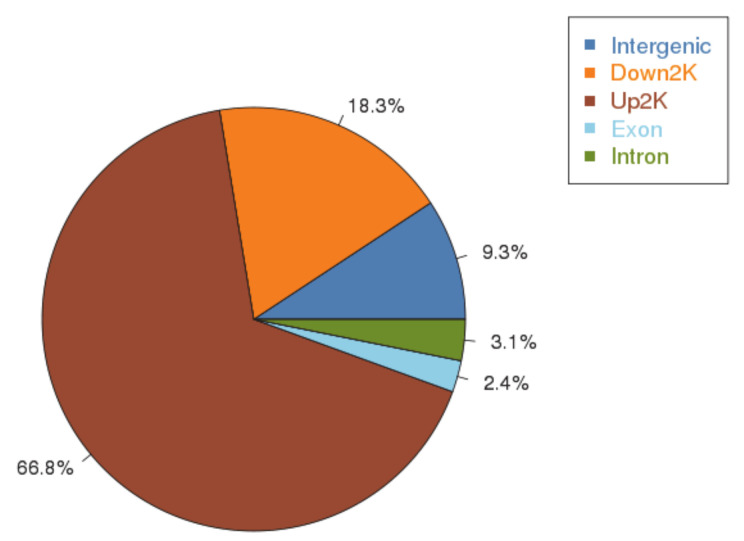
ChIP-seq peaks distribution.

To identify genome-wide transcriptional targets of AfAflR, ChIP-seq analysis was accomplished using the AflR-specific antibody against the three biological samples. The total numbers of Illumina 75-bp paired end reads used for peak calling were 1,707,715 and 9,637,795 for ChIP and the input control, respectively. Reads were aligned to the *A. flavus* NRRL3357 genome sequence and used for peak calling with the MACS2 program. A total of 540 peaks associated with AflR binding events were identified (Table S1). Their distributions are shown in Figure 2 and associated sequence information listed in Table S2. The results indicated that 66.8% of these peaks were located within 2 kb upstream (promoter region) of translational start sites (ATG) (Figure 2). 

**Figure 3:**
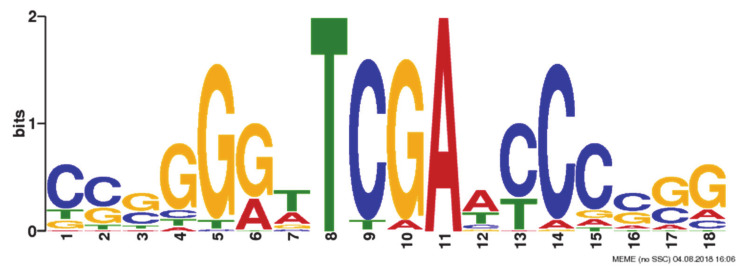
AfAflR binding motif identified using MEME.

An AflR-binding motif in the *A. flavus* genome sequence (Figure 3) was discovered by the MEME analysis. This consensus motif with an E-value of 3.1e-272 was built from the 540 peak sequences (Figure S1). The motif was an 18-bp region predominated by GGGTTCGAACCC in positions 4–15, a thymine in position 8, and an adenine in position 11. Other positions were more variable. A part (positions 8–18) of the DNA motif is similar to previously identified AflR-binding motifs of *A. parasiticus* and *A. nidulans* that were discovered using EMSA and foot-printing techniques [5,6]. Locations of motif sites identified by MEME are shown in Figure S1. We identified similar AflR motifs in promoter sequences near some aflatoxin cluster genes (see below) but did not identify actual binding events associated with aflatoxin cluster genes from the 540 peak sequences. We speculate that the expression level of aflatoxin cluster genes in this study was low.

The motif we first identified was a palindromic sequence (Figure 3). However, in comparison to those bound by AflRs of *A. parasiticus* and *A. nidulans*, we hypothesized that positions 8–18 was one motif (motif A), while positions 1–11 was another motif (motif B). Motif B was identical to motif A but was on the lower reverse complementary strand. AfAflR probably binds to either or both of the 11-bp motif A and motif B. This modification would put the AfAflR binding motif to be in line with established findings from *A. nidulans* and *A. parasiticus*. In the work of Fernandes et al. [5], although the palindromic 5′-TCG(N5)CGA-3′ was proposed from the study of the *stcU* gene promoter, partial binding of AflR to 5′-TCGga and 5′-TCGg was also found by the methylation interference foot-printing assay. In the work of Ehrlich et al. [6] consensus binding sequences, 5′-TCGSWNNSCGR-3′ (S = G/C, W = A/T, R = A/G and N = A/T/G/C) start with 5′-TCG, but they are not necessarily perfect palindromic sequences. Surprisingly, one third (6/17) of the *A. nidulans* genes involved in sterigmatocystin biosynthesis do not have the AflR-binding motif present in their promoters [5]. Similarly, only nine out of the 25 known aflatoxin biosynthetic genes in *A. parasiticus* have the AflR-binding motif identified in their promoter regions [6]. The derived motif sequence from *A. parasiticus*, however, is consistent with our conclusion of the *A. flavus* AflR-binding motif, 5′-**TCG**A**WCCCSSG**-3′ (S = G/C and W = A/T). For example, AflR of *A. parasiticus* is able to bind to 5′ TCGCAGCCCGG-3′ present in the promoter of the aflatoxin biosynthetic gene *avnA* [6], which resembles the predominant *A. flavus* AflR-binding sequence, TCGAACCCCGG (Figure 3). In this study, we found AflR of *A. flavus* preferentially bound to TCGA in vivo. In comparison, AflR of *A. nidulans* in the foot-printing experiment with the *stcU* promoter region solely binds to TCGG, but in EMSA it also binds to motifs containing TCG(C/A). Two other *A. nidulans* sterigmatocystin biosynthetic genes, *stcI* and *stcJ*, also contain in their promoters putative motifs with TCGA [5]. AflR of *A. parasiticus* mainly binds to TCG(G/C) in EMSA. However, a putative motif containing TCGA is present in the promoter of *ver1* (=*A. nidulans stcU*). Intriguingly, a few identified binding motifs despite being perfect are not recognized by *A. parasiticus* AflR; the underlying reason is not known [5,6]. This 5′-TCG likely is the canonical binding anchor for AflRs of aspergilli. An AflR-binding sequence thus is bipartite, with 5′-TCG and CGR-3′ at the front-most and distal ends, respectively, separated by a spacer of pentanucleotide. Zn(II)2Cys6 binuclear cluster transcriptional activators are believed to form dimers [23]. Likely, two AflR monomers as a functional dimer binding to both trinucleotides are required to initiate transcription. The aforementioned binding of *A. nidulans* AflR to the partial motif of 5′-TCG in the foot-printing experiment probably results from AflR monomers. Therefore, evidence obtained from the present in vivo and previous in vitro studies unambiguously supports the binding specificity of AflR to 5′-TCG. Results from this study also give support to the work of Ehrlich et al. (2012) by ChIP [24], which showed that c-Myc-tagged AflR expressed in *A. flavus* was able to pull down aflatoxin gene fragments, although the actual binding motif sequences were not revealed by their study.

**Figure 4:**
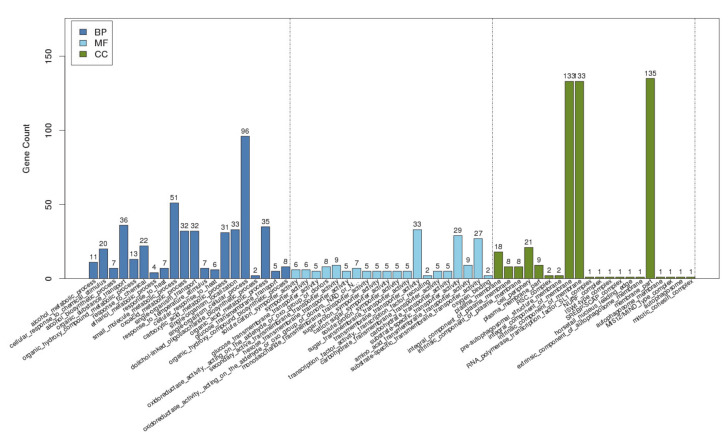
GO analysis. BP: biological process; MF: molecular function; CC: cellular component.

To further determine what other genes AflR binds to, we used gene ontology (GO) to analyze the enriched GO terms, which are classified into biological processes, molecular function, and cellular components (Figure 4). For biological processes, AfAflR mostly bound to genes related to single-organism cellular processes, small molecule metabolic processes, organic substance transport, etc. For molecular function, AfAflR mostly bound to genes related to substrate-specific transmembrane transporter activity, carbohydrate transporter activity, transmembrane transporter activity, etc. For cellular components, the results indicate that AfAflR binds near genes whose products are associated with the cell membranes. The binding of AfAflR to a diverse array of genes in the genome suggests that its binding capacity and specificity identified are genuine. Whether some of the genes to whose promoters AfAflR also bound are under the same control warrants further study. 

In conclusion, the binding motif of AfAflR was identified by ChIP-seq. As a GAL4-type regulatory protein, we believe that AflR plays an important role in gene regulation in *Aspergillus*. Examining the role of AflR outside the aflatoxin biosynthetic cluster is an ongoing research goal to understand the comprehensive functions of AflR in aspergilli.

## Supplementary Materials

The following are available online at https://www.mdpi.com/2309-608X/6/2/52/s1, Table S1: Significant peaks from AfAflR ChIP-seq, Table S2: Annotation of the peaks (AfAflR_vs_input). Figure S1: Locations of motif sites identified by MEME.

## References

[1] Flaherty J.E., Payne G.A. (1997). Overexpression of *aflR* leads to upregulation of pathway gene transcription and increased aflatoxin production in *Aspergillus flavus*. Appl. Environ. Microbiol..

[2] Yu J., Chang P.K., Ehrlich K.C., Cary J.W., Bhatnagar D., Cleveland T.E., Payne G.A., Linz J.E., Woloshuk C.P., Bennett J.W. (2004). Clustered pathway genes in aflatoxin biosynthesis. Appl. Environ. Microbiol..

[3] Chang P.K., Cary J.W., Bhatnagar D., Cleveland T.E., Bennett J.W., Linz J.E., Woloshuk C.P., Payne G.A. (1993). Cloning of the *Aspergillus parasiticus apa-2* gene associated with the regulation of aflatoxin biosynthesis. Appl. Environ. Microbiol..

[4] Payne G.A., Nystrom G.J., Bhatnagar D., Cleveland T.E., Woloshuk C.P. (1993). Cloning of the *afl-2* gene involved in aflatoxin biosynthesis from *Aspergillus flavus*. Appl. Environ. Microbiol..

[5] Fernandes M., Keller N.P., Adams T.H. (1998). Sequence-specific binding by *Aspergillus nidulans* AflR, a C6 zinc cluster protein regulating mycotoxin biosynthesis. Mol. Microbiol..

[6] Ehrlich K.C., Montalbano B.G., Cary J.W. (1999). Binding of the C6-zinc cluster protein, AFLR, to the promoters of aflatoxin pathway biosynthesis genes in *Aspergillus parasiticus*. Gene.

[7] Yu J., Butchko R.A.E., Fernandes M., Keller N.P., Leonard T.J., Adams T.H. (1996). Conservation of structure and function of the aflatoxin regulatory gene *aflR* from *Aspergillus nidulans* and *A. flavus*. Curr. Genet..

[8] Payne G.A., Nierman W.C., Wortman J.R., Pritchard B.L., Brwon D., Dean R.A., Bhatnagar D., Cleveland T.E., Machida M., Yu J. (2006). Whole genome comparison of *Aspergillus flavus* and *A. oryzae*. Med. Mycol..

[9] Nierman W.C., Yu J., Fedorova-Abrams N.D., Losada L., Cleveland T.E., Bhatnagar D., Bennett J.W., Dean R., Payne G.A. (2015). Genome sequence of *Aspergillus flavus* NRRL 3357, a strain that causes aflatoxin contamination of food and feed. Genome Announc..

[10] Price M.S., Yu J., Nierman W.C., Kim H.S., Pritchard B., Jacobus C.A., Bhatnagar D., Cleveland T.E., Payne G.A. (2006). The aflatoxin pathway regulator AflR induces gene transcription inside and outside of the aflatoxin biosynthetic cluster. FEMS Microbiol. Lett..

[11] Park P.J. (2009). ChIP-seq: Advantages and challenges of a maturing technology. Nat. Rev. Genet..

[12] Johnson D.S., Mortazavi A., Myers R.M., Wold B. (2007). Genome-wide mapping of in vivo protein-DNA interactions. Science.

[13] Liang Y., Kong Q., Yao Y., Xu S.J., Xie X. (2019). Fusion expression and anti-*Aspergillus flavus* activity of a novel inhibitory protein DN-AflR. Int. J. Food Microbiol..

[14] Signore M., Reeder K.A. (2012). Antibody validation by Western blotting. Methods Mol. Biol..

[15] Kong Q., Shan S., Liu Q., Wang X., Yu F. (2010). Biocontrol of *Aspergillus flavus* on peanut kernels by use of a strain of marine *Bacillus megaterium*. Int. J. Food Microbiol..

[16] Chung D., Barker B.M., Carey C.C., Merriman B., Werner E.R., Lechner B.E., Dhingra S., Cheng C., Xu W., Blosser S.J. (2014). ChIP-seq and in vivo transcriptome analyses of the *Aspergillus fumigatus* SREBP SrbA reveals a new regulator of the fungal hypoxia response and virulence. PLoS Pathog..

[17] Quail M.A., Kozarewa I., Smith F., Scally A., Stephens P.J., Durbin R., Swerdlow H., Turner D.J. (2008). A large genome center’s improvements to the Illumina sequencing system. Nat. Methods.

[18] Langmead B., Salzberg S.L. (2012). Fast gapped-read alignment with Bowtie 2. Nat. Methods.

[19] Zhang Y., Liu T., Meyer C.A., Eeckhoute J., Johnson D.S., Bernstein B.E., Nusbaum C., Myers R.M., Brown M., Li W. (2008). Model-based analysis of ChIP-Seq (MACS). Genome Biol..

[20] Ashburner M., Ball C.A., Blake J.A., Botstein D., Butler H., Cherry J.M., Davis A.P., Dolinski K., Dwight S.S., Eppig J.T. (2000). Gene Ontology: Tool for the unification of biology. The Gene Ontology Consortium. Nat. Genet..

[21] Alexa A., Rahnenfuhrer J. topGO: Enrichment analysis for gene ontology. R package version 2.38.1..

[22] Bailey T.L., Williams N., Misleh C., Li W.W. (2006). MEME: Discovering and analyzing DNA and protein sequence motifs. Nucleic Acids Res..

[23] Schjerling P., Holmberg S. (1996). Comparative amino acid sequence analysis of the C6 zinc cluster family of transcriptional regulators. Nucleic Acids Res..

[24] Ehrlich K.C., Mack B.M., Wei Q., Li P., Roze L.V., Dazzo F., Cary J.W., Bhatnagar D., Linz J.E. (2012). Association with AflR in endosomes reveals new functions for AflJ in aflatoxin biosynthesis. Toxins.

